# Assessing the Knowledge, Attitude, and Practice of Orthopedicians for Pain Management by Multimodal Approach: A Prospective, Cross-Sectional, and Observational Survey

**DOI:** 10.7759/cureus.59935

**Published:** 2024-05-08

**Authors:** Gurinder S Bedi, Shuvendu P Roy, Vishwadeep Sharma, Seema V Bhagat, Arti P Sanghavi, Snehal S Muchhala, Sagar Katare, Bhavesh P Kotak, Ritwik Banerjee

**Affiliations:** 1 Orthopaedics, Fortis Healthcare, New Delhi, IND; 2 Medical Affairs, Dr. Reddy's Laboratories Ltd, Hyderabad, IND

**Keywords:** multi-modality pain management, utilization, renal, pain score, gastrointestinal

## Abstract

Background: The routine use of multimodal analgesic modality results in lower pain scores with minimum side effects and opioid utilization.

Materials and methods: A prospective, cross-sectional, observational study was conducted among orthopedicians practicing across India to assess the professional opinions on using analgesics to manage orthopedic pain effectively.

Results: A total of 530 orthopedicians participated in this survey. Over 50% of the participants responded that tramadol with or without paracetamol was the choice of therapy for acute pain. Nearly 50% of the participants mentioned that multimodal interventions can sometimes help to manage pain. A total of 55.6% of participants mentioned that using Non-steroidal anti-inflammatory drugs was the most common in their clinical practice, while 25.7% of participants mentioned that they used tramadol more commonly in their clinical practice. As per clinical efficacy ranking, the combination of tramadol plus paracetamol (44.3%) was ranked first among analgesic combinations, followed by aceclofenac plus paracetamol (40.0%). The severity of pain (62.6%) followed by age (60.6%) and duration of therapy (52.6%) were the most common factors that should be considered while prescribing tramadol plus paracetamol combination. Gastrointestinal and renal are reported as the most common safety concerns encountered with analgesics.

Conclusion: The combination of tramadol and paracetamol was identified as the most preferred choice of analgesics for prolonged orthopedic pain management.

## Introduction

Pain is a global health issue affecting millions worldwide [1]. A recent study revealed that there is a notable difference in pain prevalence among countries, with rates ranging from 9.9% to 50.3% [1]. In India, pain is a major public health concern, particularly among older adults, with an estimated prevalence rate of 36.6% [2].

Pain can be classified based on several factors, including the region of the body affected, the pattern of occurrence and duration (acute or chronic), and the system or dysfunction that may be causing the pain. The pain pathway involves three main stages, beginning with pain sensitivity followed by activation of nociceptors in the peripheral nervous system. When these receptors are activated, they send signals along sensory neurons to the spinal cord. In the last stage, the signals are transmitted to the higher brain, where they are processed and interpreted as pain. The brain then sends signals back down the spinal cord to activate descending pathways that can modulate the pain signals [3]. Pain management is often complex and multifactorial, requiring a comprehensive approach that takes into account the underlying causes of the pain. One of the biggest challenges in pain management is the lack of understanding among healthcare professionals about the nature of pain, options for management, and the best approaches to managing it. This can lead to inadequate pain relief for patients, as well as unnecessary suffering and complications. To address this issue, it is important to identify the barriers that exist to proper and optimum pain management and to develop strategies to overcome them.

Guidelines for treating pain in orthopedic conditions are generally based on the principle of the World Health Organization (WHO) ladder [4]. The main pharmacological treatments for pain management include paracetamol, non-steroidal anti-inflammatory drugs (NSAIDs), and opioids. However, in some cases, a combination of analgesics may be necessary to achieve optimal pain relief. It's important to note that there is no one-size-fits-all approach when it comes to pain management, and choosing the most appropriate treatment option requires consideration of various factors, such as patient characteristics, medication-related factors, co-morbid conditions, pain etiology, and disease-specific aspects. Therefore, physicians must carefully evaluate each case and select the best-suited medication from the available options to achieve effective pain control.

Multimodal analgesic techniques involve the simultaneous administration of two or more analgesic agents targeting pain pathways to reduce the dosage and consequent side effects. This approach has gained widespread favor among perioperative healthcare professionals (HCPs) for managing osteoarthritis patients. The routine use of multimodal analgesia results in lower pain scores with fewer side effects and less reliance on opioids [5].

The prescription of NSAIDs has conventionally been the primary choice in general orthopedic conditions; however, there may be different etiology and diagnosis, and the choice of drug used could be from the plethora of analgesic options used. Furthermore, the data related to the current practice of orthopedician on the choice of drugs and prescription for the management of different painful conditions and guidance amongst orthopedician is scarce in India.

Hence, there is an unmet need to assess the professional opinions and understanding of orthopedician regarding the use of analgesics to manage orthopedic pain effectively.

## Materials and methods

This was a prospective, cross-sectional, observational study conducted in Orthopedician practicing at multiple clinics, hospitals, or colleges across various regions of India. Consulting orthopedics fulfilling participation criteria were asked to participate in the study and complete the online questionnaire. The questionnaire was shared with participants via email and/or WhatsApp.

The study was conducted in compliance with the ethical principles of the Declaration of Helsinki, ICH-GCP guidelines, and other applicable local regulatory guidelines. The trial was approved by the Independent Ethics Committee (Royal Pune Independent Ethics Committee), EC Reg. No: ECR/45/Indt/MH/2013/RR-19. The trial was prospectively registered on the Clinical Trial Registry of India (CTRI/2022/10/046503).

Both open- and close-ended questions were included in the study questionnaire. The questionnaire consisted of 22 questions across each category, as mentioned below: Understanding practice dynamics; Understanding the knowledge of analgesics and pain; Attitude toward the management of pain; Safety concerns on longer usage of analgesics; Understanding physician's practice for the management of pain by a multimodal approach.

No formal sample size calculation was carried out for this study. The study was completed with 530 complete cases. There were no dropouts. Data was analyzed for 530 participants.

Statistical analysis

Statistical analysis was carried out using the 23.0 version of the statistical software SPSS. Data describing qualitative variables were presented as counts and percentages.

## Results

A total of 530 orthopedicians participated in this survey. The majority of participants had 10-20 years of clinical practice. A total of 218 (41.1%), 147 (27.7%), 147 (27.7%), and 18 (3.5%) participants were from the Western, Southern, Northern, and Eastern parts of India, respectively.

**Figure 1:**
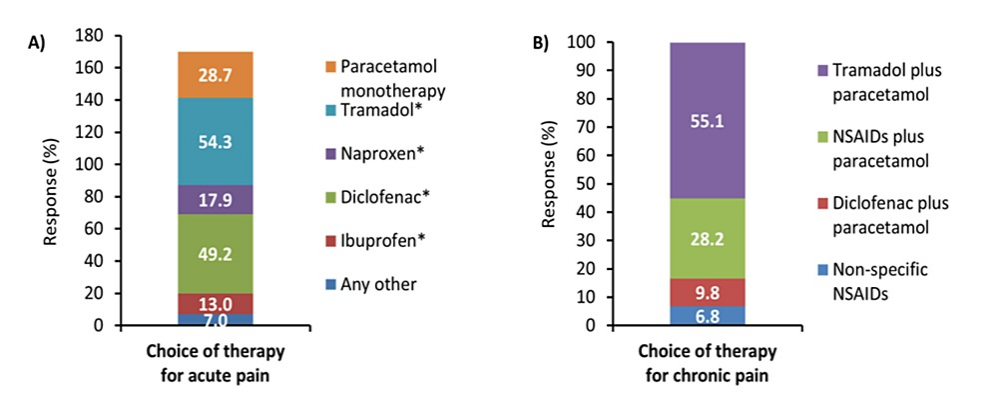
Response for questions A) What is your choice of therapy for acute pain? B) What is your choice for chronic pain? (N=530) Data presented as %. NSAIDs: Non-steroidal anti-inflammatory drugs

Around 95% of participants opined that they used different treatment strategies for acute pain and chronic pain. More than 50% of the participants responded that tramadol with or without paracetamol was the choice of therapy for acute pain (Figure 1A). For chronic pain, 55.1% of participants selected tramadol plus paracetamol as the choice of therapy (Figure 1B).

**Figure 2:**
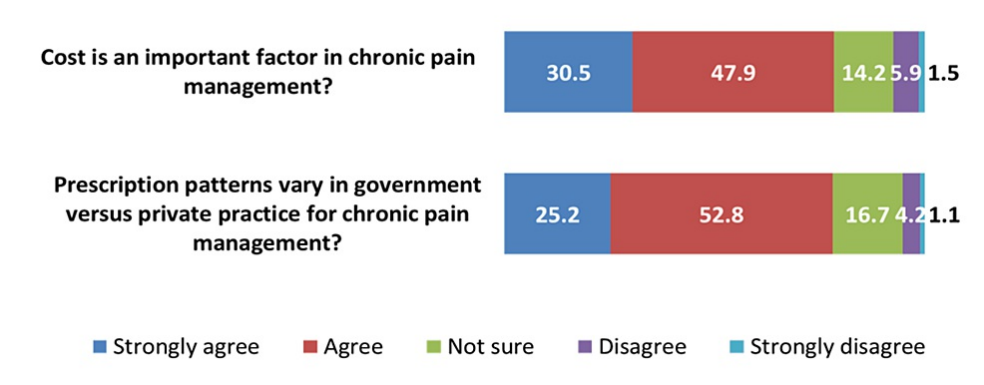
How much do you agree that cost is an important factor in chronic pain management? (N=528) and prescription patterns vary in government versus private practice for chronic pain management? (N=527) Data presented as %.

Considering the management of chronic pain, 78.4% of participants agreed or strongly agreed that cost is an important factor, and 78.0% of participants agreed or strongly agreed that prescription patterns vary in government and private practice (Figure 2).

Most participants (57.3%) responded that Indian Ortho's practice guidelines for pain management are required. Around 50% of the participants mentioned that multimodal interventions can sometimes help to manage pain (Table 1).

**Table 1:** Response to survey questions

Questions	Opinion options	Percentage of doctor's response
What is your opinion on guidelines for ortho-practice-related pain management? (N=518)	Current guidelines are sufficient	128 (24.7)
	Indian Ortho’s practice guidelines for pain management are required	297 (57.3)
	Clinical experience is enough, as guidelines are not practical	89 (17.2)
	I follow specific guidelines, such as	4 (0.8)
	Specified (N=2)	
	Specific guidelines in my hospital	1 (0.2)
	Follow clinical experience and change as per the patient	1 (0.2)
What do you think about the role of multimodal interventions in your practice? (N=520)	Not much/Rarely preferred in my practice	77 (14.8)
	Sometimes it helps	262 (50.4)
	Often it helps the patient	93 (17.9)
	Very helpful to the patient	88 (16.9)
What are newer agents used by you in practice? (N=514)	Fentanyl patch	68 (13.2)
	Tapentadol	145 (28.2)
	Flupiritine	57 (11.1)
	Buprenorphine	75 (14.6)
	Ketoprofen/Diclofenac patch	139 (27.0)
	Lignocaine patch	30 (5.8)
Do you prescribe anti-depressant and anti-anxiety classes of medication to manage pain with psychological concerns? (N=524)	Yes, they are often required to be prescribed	130 (24.8)
	Sometimes, as per the patient's profile	280 (53.4)
	Not at all, as pain medication is sufficient	76 (14.5)
	It’s better to refer them to a Neuro or Psychiatry specialist	38 (7.3)
	Any other (not specified)	1 (0.2)

A total of 55.6% of participants mentioned that the use of NSAIDs was the most common in their clinical practice. In comparison, 25.7% of participants mentioned that they used tramadol more commonly in their clinical practice. The majority of participants rated rheumatoid arthritis as the most common orthopedic condition that required prolonged usage of analgesics (42.1% of doctors rated it as rank 1). In addition, knee osteoarthritis was the second most common orthopedic condition requiring prolonged analgesic use (Table 2).

**Table 2:** In your clinical experience, which is the major orthopedic condition requiring/demanding prolonged usage of analgesics?

Orthopedic condition	Rank						
	1	2	3	4	5	6	7
Low back pain (N=233)	80 (24.3)	54 (23.2)	49 (21.0)	12 (5.2)	28 (12.0)	6 (2.6)	4 (1.7)
Knee osteoarthritis (N=241)	68 (28.2)	98 (40.7)	33 (13.7)	16 (6.6)	14 (5.8)	7 (2.9)	5 (2.1)
Rheumatoid arthritis (N=252)	106 (42.1)	39 (15.5)	46 (18.3)	30 (11.9)	19 (7.5)	7 (2.8)	5 (2.0)
Neck and shoulder pain (N=202)	15 (7.4)	26 (12.9)	50 (24.8)	77 (38.1)	25 (12.4)	4 (2.0)	5 (2.5)
Elbow pain (N=164)	10 (6.1)	8 (4.9)	10 (6.1)	35 (2.1)	93 (56.7)	5 (3.0)	3 (1.8)

Patients' compliance (37.9%) was reported as the most common reason for poor control of postoperative pain, followed by fragmentation of care/lack of clear standards and patient expectations (28.5%). A total of 68.0% of participants agreed or strongly agreed that NSAIDs have greater safety concerns when compared to tramadol plus paracetamol when used even for a shorter duration (Table 3).

**Table 3:** Response to survey questions NSAIDs: Non-steroidal anti-inflammatory drugs

Questions	Opinion options	Percentage of doctor's response
What are the reasons for poor control of postoperative pain? (N=519)	Inadequate opioid prescribing	81 (15.6)
	Fragmentation of care/lack of clear standards and patient expectations	134 (25.8)
	Patients compliance	197 (37.9)
	Lack of pre-operative analgesia	74 (14.3)
	Poor prescription practice	28 (5.4)
	Any other	5 (1.0)
	Specified (N=1) Proper balance between NSAIDs and advice followed by patient	1 (0.2)
In your clinical practice, how often do you involve cross-specialty experts (psychiatrists, occupational therapists, anesthetists, etc)? (N=521)	<10%	181 (34.7)
	10-20%	154 (29.6)
	20-30%	125 (24.0)
	30-50%	41 (7.9)
	>50%	20 (3.8)
Do you believe that NSAIDs have greater safety concerns when compared to Tramadol plus Paracetamol when used even for a shorter duration? (N=518)	Strongly agree	92 (17.8)
	Agree	260 (50.2)
	Not sure	99 (19.1)
	Disagree	62 (11.9)
	Strongly disagree	5 (1.0)

According to the clinical efficacy ranking, the combination of tramadol plus paracetamol (44.3%) was ranked first among analgesic combinations, followed by aceclofenac plus paracetamol (40.0%) (Table 4).

**Table 4:** As per the clinical efficacy, rate the following analgesic combinations.

Analgesic combinations	Rank				
	1	2	3	4	5
Tramadol + Paracetamol (N=291)	129 (44.3)	59 (20.3)	38 (13.1)	25 (8.6)	40 (13.7)
Tramadol + Diclofenac (N=140)	23 (16.4)	36 (25.7)	30 (21.4)	32 (22.9)	19 (13.6)
Nimesulide + Paracetamol (N=129)	5 (3.9)	13 (10.1)	31 (24.0)	28 (21.7)	52 (40.3)
Aceclofenac + Paracetamol (N=208)	79 (40.0)	60 (28.8)	27 (12.9)	18 (8.7)	24 (11.5)
Ibuprofen + Paracetamol (N=141)	6 (4.3)	25 (17.7)	43 (30.5)	41 (29.1)	26 (18.4)

The severity of pain (62.6%) followed by age (60.6%) and duration of therapy (52.6%) were the most common factors that should be considered while prescribing tramadol plus paracetamol combination. For patients with chronic pain, the perception of tramadol plus paracetamol in terms of efficacy and tolerability was generally positive. Specifically, 20.4% of participants reported being very satisfied, and 60.6% reported being satisfied (Table 5).

**Table 5:** Response to survey questions COX: Cyclooxygenase

Questions	Opinion options	Percentage of doctor's response
What are the factors to be considered when prescribing Tramadol plus Paracetamol? (N=530)	Age	321 (60.6)
	Severity of pain	332 (62.6)
	Duration of therapy	279 (52.6)
	Frequency of dosing	183 (34.5)
	Dosing interval	121 (22.8)
With your clinical experience, kindly rate the perception of patients with chronic pain about Tramadol plus Paracetamol based on efficacy and tolerability (N=515)	Very satisfied	105 (20.4)
	Satisfied	312 (60.6)
	Neutral	75 (14.6)
	Unsatisfied	15 (2.9)
	Very Unsatisfied	8 (1.6)
What is the longest duration of prescription for Tramadol + Paracetamol used in your clinical practice? (N=518)	Less than 2 weeks	173 (33.4)
	2 to 4 weeks	233 (45.0)
	1 to 3 months	76 (14.7)
	3 to 6 months	26 (5.0)
	> 6 months	10 (1.9)
Do you find the difference between selective and non-selective COX inhibitors in ortho practice? (N=515)	Yes	379 (73.6)
	No	84 (16.3)
	Don’t know	52 (10.1)

**Figure 3:**
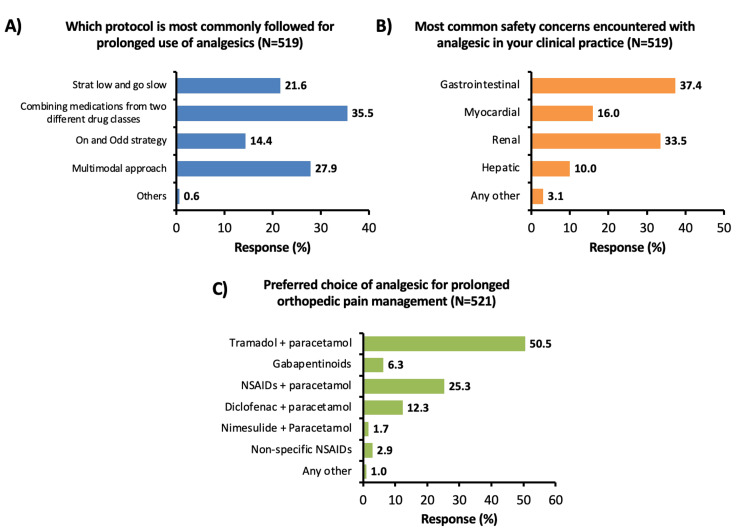
Doctor's response to question A) In your clinical experience, which is the most commonly followed protocol for prolonged use of analgesics? B) Which are the most common safety concerns encountered with analgesic use in your practice C) Which is the preferred choice of analgesic for prolonged orthopedic pain management? NSAIDs: Non-steroidal anti-inflammatory drugs

A total of 35.5% of participants mentioned that combining medications from two different medical classes was the most commonly followed protocol for prolonged use of analgesics (Figure 3A). Gastrointestinal (GI) and renal are reported as the most common safety concerns encountered with analgesics (Figure 3B). Among analgesics, tramadol in combination with paracetamol was chosen by 50.5% of participants as the most preferred choice of analgesic for prolonged orthopedic pain management (Figure 3C).

## Discussion

In India, the management of acute and chronic pain involves a combination of pharmacological and non-pharmacological therapies. The approach to pain management in India is generally conservative, with opioids being used only when other treatments have failed. For acute pain management, non-opioid analgesics such as paracetamol and NSAIDs are commonly used [6]. For chronic pain management, opioids are not the first line of treatment. Instead, non-opioid analgesics like paracetamol and NSAIDs, as well as adjuvant drugs like anti-depressants, anti-convulsants, and muscle relaxants, are used [7]. In recent years, there has been an increasing emphasis on the use of interventional pain management techniques like nerve blocks and epidural injections for both acute and chronic pain management. In addition, the government of India has implemented the National Programme for Palliative Care to improve access to palliative care and pain management for patients with advanced illnesses [8]. It is important to note that the management of pain in India may vary depending on the severity of pain and the availability of resources.

In the present study, participants opined that they used different treatment strategies for acute and chronic pain, where tramadol with or without paracetamol was the most commonly prescribed analgesic for both acute and chronic pain, respectively. In contrast, Billa et al. reported the practice of pain management by Indian healthcare practitioners where 88.64% of orthopedicians opted for non-specific NSAIDs to treat mild pain, while 80.30% of them favored the combination of tramadol and paracetamol for moderate pain. Meanwhile, combinations of NSAID and paracetamol and paracetamol plus diclofenac were preferred by 68.94% and 47.73% of orthopedicians, respectively, for managing moderate pain [9]. Here, the current and previous studies appear to have different treatment strategies. This may be due to changes in medical practices, differences in study design, regional variations, and changes in drug availability. Moreover, around half of the participants indicated a requirement for pain management guidelines specific to Indian orthopedic practices.

In an effort to decrease opioid dependency, HCPs are turning to multimodal pain management modalities. Based on the results of a paper-based questionnaire survey, most HCPs suggested multimodal analgesia modality for better pain management [9]. Similarly, a 10-year database review from 2006 to 2016 of total hip and knee arthroplasties (N=1,540,462) showed that the majority of patients (85.6%) received multimodal analgesia, which resulted in improved patient outcomes with decreased opioid dependency [10]. However, in the present study, half of the participants mentioned that multimodal interventions sometimes help to manage pain in their clinical practice. This explains that the optimal multimodal regimen is still unknown; these findings encourage the promotion of multimodal treatment protocols for managing pain.

It is interesting to note that according to the participants' opinions, rheumatoid arthritis, and knee osteoarthritis are the most common orthopedic conditions that require prolonged use of analgesics. This could be due to the chronic nature of these conditions, which often lead to persistent pain and discomfort. On the other hand, low back pain and elbow pain were perceived as requiring the least amount of prolonged analgesic use by doctors.

Being used widely and frequently, analgesics are often associated with adverse drug reactions. The main safety concerns with using analgesics are GI complications, renal, cardiovascular, hematologic effects, and hepatic and allergic reactions [11]. In the present study, the majority of participants reported analgesics associated with renal and hepatic adverse events. Similarly, previously published studies exploring the risk of GI bleeding among analgesic users systematically reported a positive association with GI bleeding [12,13]. Regarding renal outcomes, Zhang et al. demonstrated that the use of NSAIDs increased the risk of kidney injury in patients with chronic kidney disease. This relationship was also confirmed in a more recent study by Chiasson et al. [14,15].

Tramadol is superior to chronic NSAIDs due to its lower incidence of drug-induced GI and renal toxicity. Interestingly, a significant proportion of participants believe that NSAIDs may have greater safety concerns than tramadol, even with a shorter duration of use. Various studies have shown that tramadol exerts less GI toxicity than NSAIDs [16]. In contrast, a meta-analysis study comparing the pain reduction ability of oral NSAIDs and opioids for knee osteoarthritis noted a higher proportion of GI-related adverse events with tramadol than with NSAIDs [17]. Moreover, it remains unclear whether the use of tramadol is a safer analgesic for patients with GI disorders.

The majority of participants reported considering the severity of pain, age, and duration of therapy when prescribing the combination of tramadol plus paracetamol, with the longest duration of prescription of 2 to 4 weeks.

Recent trends in chronic pain medicine have highlighted the growing interest in non-pharmacological approaches to pain management. Mindfulness-based stress reduction (MBSR) programs have gained popularity as a complementary therapy for chronic pain patients. Studies have shown that participation in MBSR programs can significantly reduce pain severity and improve overall well-being [18]. In this study, researchers compared the effectiveness of MBSR with traditional pain management approaches in patients with chronic low back pain [18]. The results demonstrated that MBSR not only reduced pain but also enhanced patients' ability to cope with pain-related distress and disability. This approach aligns with the broader shift in healthcare toward holistic and patient-centered care.

In contrast, the present study's findings underscore the continued preference for a multimodal approach that combines various pharmacological therapies to manage chronic pain. The study reveals a reliance on medications like tramadol, paracetamol, and NSAIDs, reflecting the continued importance of pharmacological agents in pain control strategies. This approach is consistent with the broader medical paradigm that acknowledges the efficacy of pharmaceuticals in pain relief.

Some of the key barriers to effective pain management include a lack of education and training among healthcare professionals. Inadequate access to pain medications and other treatments and cultural and social attitudes that stigmatize pain and discourage patients from seeking help are the other concerns. To overcome these barriers, healthcare organizations and policymakers need to invest in education and training programs for healthcare professionals. Improving access to medications and pain management modalities and promoting awareness and understanding of pain as a legitimate health issue need to be taken up as a priority.

## Conclusions

Overall, the study's findings suggest a shift in orthopedic pain management practice over the years towards a multimodal analgesic approach involving tramadol and paracetamol as the preferred choice while being mindful of safety concerns associated with NSAIDs. Orthopedicians should consider adopting this approach as it offers a more effective and safer way to manage pain in their patients. Moreover, the findings underscore the importance of understanding and addressing the diverse treatment strategies employed in orthopedic pain management, highlighting the need for tailored guidelines to optimize patient care. Continuing research and collaboration within the orthopedic community are essential to refine and improve analgesic strategies, ultimately enhancing patient outcomes and quality of care.
